# Inhibition of α-Amylase and α-Glucosidase Using *Baccaurea ramiflora*

**DOI:** 10.1155/sci5/8768659

**Published:** 2025-06-16

**Authors:** Pongsathorn Klomsakul, Pornchanok Chalopagorn

**Affiliations:** ^1^Department of Biology, Faculty of Science and Technology, Phranakhon Rajabhat University, Bangkok 10220, Thailand; ^2^Department of Chemistry, Faculty of Science and Technology, Phranakhon Rajabhat University, Bangkok 10220, Thailand

**Keywords:** α-amylase, α-glucosidase, *Baccaurea ramiflora* extracts, diabetes

## Abstract

The hunt for alternative antidiabetic treatments that result in fewer side effects judged against established treatments is being powered by the mounting worldwide pervasiveness of diabetes mellitus. Playing a critical role in many biological processes, *Baccaurea ramiflora*, a long-established medicinal plant commonly used throughout Southeast Asia, presents abundant health benefits. An assessment of the various ethanol extracts from *B. ramiflora* drawn from the leaves, stems, stem bark, peel, and flesh with seed revealed the total phenolic and flavonoid contents in addition to the levels of inhibitory activity against the enzymes α-amylase and α-glucosidase. In the case of total phenolic and flavonoids contents, the findings confirmed that the leaves, flesh with seed, and peel extracts were the richest source of phenolics. The leaf extract showed higher flavonoid content than others. The greatest levels of inhibitory activity against the two enzymes of interest were exhibited by the flesh with seed and peel: the respective IC_50_ values associated with α-amylase and α-glucosidase were 0.50 ± 0.03 and 0.69 ± 0.01 mg mL^−1^ for flesh with seed, and 0.61 ± 0.04 and 0.59 ± 0.01 mg mL^−1^ for peel. The various extracts were all capable of inhibiting enzyme activity in a concentration-dependent manner. GC-MS analysis of the flesh with seed extract identified phenol, 3,5-bis(1,1-dimethylethyl)-, dimethylsulfoxonium formylmethylide, methyl salicylate, and other compounds. Additionally, the major compounds present in the peel extract were 2,4-di-tert-butylphenol, dimethyl sulfone, cyclooctasiloxane, hexadecamethyl, and other compounds. From these findings, *B. ramiflora* offers potential for use in the field of nutraceuticals and functional foods and might also find application in treating health conditions such as diabetes.

## 1. Introduction

Diabetes is recognized by the International Diabetes Federation (IDF) as a growing global health crisis due to the fact that this disease has over 537 million sufferers globally. This figure is anticipated to increase to 643 million by 2030, and 783 million 20 years from now [1]. Thus, diabetes mellitus is considered one of the fastest growing noncommunicable diseases (NCDs). The condition damages glucose metabolism in the body and leads to chronic hyperglycemia, in turn causing harm to different bodily organs including the nerves, kidneys, blood vessels, and eyes [2]. The management of diabetes may comprise a number of integrated techniques which include adjustments to lifestyle factors, medical treatments, and ongoing observation of symptoms. It has been shown that diabetes can be treated by targeting α-glucosidase and α-amylase, which perform the hydrolysis of carbohydrates, and many antidiabetic drugs use this approach. These key enzymes are located within the brush border layer of the small intestine, and their primary function involves the hydrolysis of long-chain dietary carbohydrates. This results in the production of monosaccharide units which can cause hyperglycemia when they enter the bloodstream [3, 4]. Postprandial hyperglycemia can be treated using synthetic inhibitors, such as miglitol, acarbose, and voglibose [5], but the use of these inhibitors presents problems in the form of undesirable side effects, which include flatulence and diarrhea, as well as liver problems and abdominal cramps [6]. These concerns have compelled researchers to search for an alternative approach which can provide effective treatment in the absence of adverse side effects. Medicinal plants are one area of interest for researchers due to their strong potential in the area of antidiabetic therapies. Indeed, medicinal plants are finding increasing usage in the treatment of disease worldwide today.

*Baccaurea ramiflora* is a member of the Phyllanthaceae family and grows throughout Southeast Asia. Traditional medical practitioners in the region have long made use of the various components of the plant [7]. As a whole plant, it is used to treat rheumatoid arthritis, as well as abscesses, cellulitis, stomach aches and ulcers, and also colic. The stem bark is known to be a diuretic, while the fruit juice can be used to treat snake bites. The seeds can be used to treat diarrhea. In addition, some natural components of *B. ramiflora* have demonstrated excellent antioxidant properties [8], as well as antibacterial activity [9, 10], anti-inflammatory and diarrheal activities [11], hypoglycemic effects [12], antihyperlipidemic activity [12, 13], analgesic properties [14], anthelmintic and insecticidal activity [15], and hepatoprotective activity [16]. The nutritional and phytochemical properties of *B. ramiflora* have been evaluated, demonstrating its nutritional value and medicinal properties. It was found that *B. ramiflora* contains important nutrients such as vitamin C, proteins, and various minerals and also includes a wide range of phytochemicals such as alkaloids, saponins, phenols, steroids, flavonoids, and fixed oil, which possess antioxidant, anti-inflammatory and antibacterial properties [17, 18]. Additionally, *B. ramiflora* contains various phytochemicals whose composition varies depending on the exact component of the plant from which they were sourced [19]. Examination of these various plant parts has been valuable in leading to different biologically active compounds being identified and used in the production of drugs [20]. However, insufficient work has been done in assessing the biological properties of some of the constituents of *B*. *ramiflora*. This study therefore seeks to establish the total phenolic and flavonoid contents of *B*. *ramiflora,* as well as determining the inhibitory capabilities against the key enzymes found in the ethanolic extracts of the different plant parts. Bioactive compounds can be identified using gas chromatography interfaced with mass spectrometry (GC-MS) analysis.

## 2. Materials and Methods

### 2.1. Plant Material

Various *B. ramiflora* parts were gathered from Nakhon Nayok, Thailand, in April 2023. The identification of *B. ramiflora* was carried out by the Biology Program at the Faculty of Science and Technology of Phranakhon Rajabhat University. Meanwhile, specimens of *B. ramiflora* (BK084916 and BKs 01741) were placed in the Bangkok Herbarium, Plant Varieties Protection Office, Bangkok, Thailand. The parts were subsequently dried in an oven for 2 days using hot air at 50°C, whereupon they were cut up and ground in a blender to produce a very fine powder. The various samples of stems, leaves, stem bark, peel, and flesh with seed were extracted in a 1-h process via maceration using 95% ethanol and an ultrasonicator. This procedure was carried out two times, whereupon the resulting extract underwent filtration using Whatman filter paper No. 1 (110 mm) before being placed in a centrifuge for 15 min at 3500 × g (Centrifuge 5810R, Eppendorf AG, Hamburg, Germany). Evaporation drying of the extract was subsequently performed using a vacuum, whereupon the extract was placed into storage at 4°C until required for further examination.

### 2.2. Total Phenolics

To calorimetrically assess the total phenolic content, the Folin–Ciocalteu procedure was utilized [21]. This required the addition of 0.1 mL of extract to 2.5 mL of Folin–Ciocalteu reagent, followed by the addition of 1 mL of an aqueous 14% Na_2_CO_3_ solution. This mixture underwent stirring and incubation for 30 min at a temperature of 40°C. A spectrophotometer (ThermoScientific UV–visible, Madison, WI, USA) was then used to measure the absorbance at 760 nm. Gallic acid served as the standard upon which the regression equation of calibration curve was based (*Y* = 0.192*x* + 0.112; *R*^2^ = 0.991), and results were presented in the form of mg of gallic acid equivalents (GAE) g^−1^ of extract.

### 2.3. Total Flavonoids

To determine the total flavonoid content [22], a mixture containing 1 mL of extract solution and 1 mL of 2% aluminum chloride (AlCl_3_) in ethanol was produced. After 10 min, the absorption at 435 nm was evaluated by UV/vis spectrophotometer to draw comparisons to a blank sample, which comprised 1 mL of the extract solution in the absence of aluminum chloride. A standard curve based on rutin (*Y* = 0.008*x* + 0.082; *R*^2^ = 0.992) served to assess the total flavonoid content by taking mean values drawn from three measurements and presenting the result in the form of rutin equivalents (RE) g^−1^ of extract.

### 2.4. Phytochemical Qualitative Analysis

Various techniques described as follows were employed to conduct the phytochemical analysis of *B. ramiflora* [23].

#### 2.4.1. Steroid Test

Concentrated sulfuric acid (2 mL) was added to acetic anhydride (2 mL) and introduced to the sample (0.5 g). Steroids were deemed to be present upon the production of a change to a green or blue color.

#### 2.4.2. Flavonoid Test

The sample (1 g) was added to ethyl acetate (10 mL) and boiled for several minutes prior to filtration. The filtrate (5 mL) was then mixed with 10% ammonia solution (1 mL) and shaken vigorously. Flavonoids were deemed to be present upon the production of a yellow color.

#### 2.4.3. Tannin Test

The sample (0.5 g) was boiled in 10 mL of distilled water prior to filtration. This was followed by the addition of several drops of 0.1% ferric chloride. The presence of tannins was indicated by brown-green or blue-black coloration.

#### 2.4.4. Terpenoid Test

The aqueous sample (5 mL) had chloroform (2 mL) introduced, followed by concentrated sulfuric acid (2 mL). Terpenoids were deemed to be present upon the production of a red-brown color.

#### 2.4.5. Anthraquinone Test

The sample (0.5 g) was mixed with chloroform (5 mL), shaken for 5 min, and then filtered, before adding 10% ammonium solution for further shaking. Anthraquinones were deemed to be present upon the production in the aqueous layer of a bright pink color.

#### 2.4.6. Reducing Sugar Test

The sample (1 g) was added to distilled water (10 mL) before being boiled and filtered, whereupon several drops of both 20% sodium hydroxide solution and Benedict solution were introduced. The resulting mixture was boiled for a further 3 min. Reducing sugars were deemed to be present upon the production of a brick red color.

#### 2.4.7. Saponin Test

The sample (1 g) was added to distilled water (10 mL) before being boiled and filtered, whereupon more distilled water (5 mL) was added for vigorous shaking. Several drops of olive oil were introduced before further shaking. Saponins were deemed to be present upon the production a foam-like appearance.

### 2.5. Analysis of α-Amylase Inhibition

The assay to assess the inhibition of the enzyme α-amylase was performed as explained earlier [24]. The initial preparation of porcine pancreatic α-amylase was performed with 0.5 mg mL^−1^ in a 20 mM sodium phosphate buffer maintaining the pH value at 6.9. Before incubation, the mixture comprised 50 μL of *B. ramiflora* extract and 50 μL α-amylase. This mixture underwent 10-min incubation at 25°C, whereupon 50 μL of a 0.5% starch solution in 20 mM sodium phosphate buffer (pH 6.9) was introduced for a further incubation period of 10 min at 25°C. To halt the reaction required the addition of 50 μL of 96 mM 3,5-dinitrosalicylic acid, whereupon the mixture underwent incubation for 10 min using heated water. A spectrophotometer (Victor Nivo, PerkinElmer, Massachusetts, USA) was employed to evaluate the solution absorbance at 540 nm. Preparation of the control followed an identical approach using a buffer in place of the extract, while the positive control was acarbose. The IC_50_ values were then calculated. The findings for α-amylase inhibition percentage are presented in the form of

(1) $$\begin{array}{c} \%\text{ inhibition}=\left[\frac{\left(A_{\text{control}}-A_{\text{sample}}\right)}{A_{\text{control}}}\right]\times 100. \end{array}$$

### 2.6. Analysis of α-Glucosidase Inhibition

Determination of α-glucosidase inhibition was performed via the assay previously mentioned [25], whereby α-glucosidase was prepared from a *Saccharomyces cerevisiae* solution involving 1 U ml^−1^ in a 20 mM sodium phosphate buffer with the pH value maintained at 6.9. Prior to incubation, this mixture comprised 25 μL of *B. ramiflora* extract and 50 μL α-glucosidase. The incubation period was 10 min at a temperature of 25°C. The next step involved the addition of 5 mM p-nitrophenyl-α-D-glucopyranoside solution in 20 mM sodium phosphate buffer at pH 6.9 (25 μL) to the mixture for 5 min of further incubation at 25°C. Spectrophotometry (Victor Nivo, PerkinElmer, Massachusetts, USA) was carried out for absorbance measurement at 405 nm. Preparation of the control involved replacement of the extract with a buffer, while acarbose was used as the positive control. The IC_50_ values obtained indicated the concentration required in order to achieve 50% inhibition. The findings for α-glucosidase inhibition percentage are presented in the form of

(2) $$\begin{array}{c} \%\text{ inhibition}=\left[\frac{\left(A_{\text{control}}-A_{\text{sample}}\right)}{A_{\text{control}}}\right]\times 100. \end{array}$$

### 2.7. GC-MS Analysis

GC-MS allowed identification of the phytochemical compounds in the ethanol extract of *B. ramiflora* peel, and flesh with seed. An Agilent 7890A-5975C Inert MSD with Triple-Axis detector was used to carry out the analysis, equipped with an Agilent 19091S-433HP-5 ms (30 m × 250 × 0.25 μM) fused silica column. Helium served as the carrier gas with the flow rate of 1 mL/min. The ion source temperature was 230°C, while the interface temperature was 325°C at a pressure of 6.6018 psi. The 1 μL injector in splitless mode had an injection temperature of 280°C. The starting temperature of the oven was 32°C, whereupon it rose by 5°C every minute up to a temperature of 325°C, after which there were no changes for 5 min. The overall time taken for the GC-MS process was 59.6 min. A relative percentage was determined for each of the components through a comparison of the mean peak area with the overall peak area [26].

### 2.8. Data Analysis

This stage involved comparisons of the average values ± standard deviation for three independent trials in each scenario. Variance analysis was then used to establish any significant differences arising between the activity levels of the various samples. A value of *p* ≤ 0.05 was considered significant.

## 3. Results and Discussion

### 3.1. Total Phenolics and Flavonoids

The influence of extraction upon the phytochemical constituents of *B. ramiflora* was evaluated using the total phenolic and flavonoid contents' assay, with outcomes presented in Table 1. To determine the total phenolic content for each of the different plant parts of *B. ramiflora*, the Folin–Ciocalteu reagent technique was utilized with results shown as GAE on the basis of a standard curve. Total phenolic content ranged from 12.33 ± 1.59 mg to 163.89 ± 26.73 mg GAE g^−1^ extract. Among the various components, the leaves (163.89 ± 26.73 mg GAE g^−1^ extract), peel (155.73 ± 11.77 mg GAE g^−1^ extract), and flesh with seed (145.83 ± 13.81 mg GAE g^−1^ extract) were the richest sources of phenolics, with nonsignificant differences between them. In contrast, the stem bark (15.10 ± 3.42 mg GAE g^−1^ extract) and the stems (12.33 ± 1.59 mg GAE g^−1^ extract) exhibited the lowest phenolic content, also with nonsignificant differences. For the determination of the flavonoid contents for each of the various parts of the *B. ramiflora* plant, the assay drew comparisons to standard RE. The results for the flavonoid contents lay in a range from 6.58 ± 1.43 to 62.55 ± 3.11 mg rutin equivalence g^−1^ extract, with the various extracts arranged from strongest to weakest: leaves (62.55 ± 3.11 mg rutin g^−1^ extract), flesh with seed (23.46 ± 2.47 mg rutin g^−1^ extract), peel (13.17 ± 2.57 mg rutin g^−1^ extract), with significant differences; and stem bark (6.99 ± 1.89 mg rutin g^−1^ extract) and stems (6.58 ± 1.43 mg rutin g^−1^ extract), with nonsignificant differences between them. However, these findings for total phenolic and flavonoid contents may differ since the values are affected by factors such as the particular plant component tested, the choice of solvent to obtain the extracts, the technique applied for analysis, environmental stress, and variations in both climate and geography [27, 28]. The various plant parts of *B. ramiflora* possess a significant F/P ratio, with the stems found to be rich in flavonoids, followed by the F/P ratios of the other extracts in descending order: stem, stem bark, leaves, flesh with seeds, and peel (Table 1). The proportion of flavonoids within the total polyphenol group is crucial for understanding how a specific quantity of flavonoids can enhance biological activity [29]. Flavonoids are the most abundant group of phenolic compounds which occur in nature, and can be observed in many different plant parts [30]. They exhibit various biochemical properties, including antioxidant, antimicrobial, and anticancer activities, as well as enzyme inhibition.

The phytochemical components of methanol, extract of seed, pulp, and peel of *B. ramiflora* were previously examined by Uddin et al., which showed the highest intensities of phenolic and flavonoid compounds were located in the pulp extract, with values of 124.360 ± 2.078 mg GAE g^−1^ and 107.527 ± 1.900 mg QRE g^−1^ extract, respectively. In contrast, the seed extract exhibited the lowest levels at 12.96 ± 0.77 mg GAE g^−1^ and 6.94 ± 0.65 mg QRE g^−1^ [31], whereas the peel extract comprised 47.23 ± 3.70 mg GAE g^−1^ and 20.72 ± 1.23 mg QRE g^−1^. Correspondingly, the influences of diverse solvents on the yield, total phenolic content, total flavonoid content, and total carotene content of *B*. *angulata* fruit were assessed by Ahmed et al. The pulp was shown to generate the greatest yield, and methanol extracts exhibited considerably greater phenolic, flavonoid, and carotene contents (*p* < 0.01) compared to those extracted with phosphate-buffered saline (PBS) across all components of fruit, as revealed by the results [32]. Moreover, as solvents for the removal of phenolic chemicals, methanol and ethanol have demonstrated efficacy [33]. The quantity and configuration of phenolic and flavonoid compounds is differentiated at the subcellular level and inside plant tissues, as verified by numerous previous studies [27]. Additionally, various sections of the same plant may produce and store distinct chemicals or varying quantities of a certain compound as a result of variable gene expression, which subsequently influences the biological characteristics of the plant extracts [34].

### 3.2. Phytochemical Analysis

In order to determine the presence of phytochemicals which might prove medicinally valuable, the phytochemical analysis was carried out to examine all of the various parts of the *B. ramiflora* plant (Table 2). The findings confirmed that flavonoids and terpenoids were present in each of the various parts. In addition, the leaves, peel, and flesh with seed were found to contain reducing sugars, while the leaves, stems, and stem bark were shown to contain tannins, and the peel contained anthraquinones. No parts of the *B. ramiflora* plant were found to contain either saponins or steroids. The initial phytochemical sampling of the genus Baccaurea [7] revealed the existence of numerous bioactive materials including terpenoids, glycosides, reducing sugar, phenols, flavonoids, alkaloids, phenols, tannins, steroids, flavonoids, volatile oils, quinones, coumarins, carbohydrates, anthraquinones, polyphenols, ascorbic acids, phytosterols, gums and mucilage, saponins, proteins and fixed oils, phlobatannin, resins, organic acids, carotenoids from different crude alcohol and aqueous extracts of *B. courtallensis*, *B. ramiflora*, *B. motleyana*, *B. lanceolata*, *B. macrocarpa*, and *B. angulate*. Plants commonly contain natural phytochemicals which offer varied biological properties and may find valuable uses in promoting human health. Among these phytochemicals, the most diverse class is that of the terpenoids, which play various roles including serving as hormones, light-harvesting pigments, semichemicals, and phytoalexins [35]. Earlier studies have reported that some herbs are the source of terpenoids which can stimulate the secretion of insulin from β-cells or can mimic the action of insulin [36]. Along with strong hypoglycemic properties, flavonoids deliver antihistamine, antiviral, anticancer and anti-inflammatory effects [37]. They also act as antioxidants. Meanwhile, tannins can reduce ROS and prevent their proliferation [38], while the varied properties of anthraquinones include antiviral, antimicrobial, antiplasmodium, and anticancer activities along with the inhibition of enzymes, stimulation of the immune system, and antiplatelet aggregation [39]. Finally, the role of sugar is crucial in plants as not only does it serve as a nutrient, but it can also support the central signaling or regulatory molecules which control plant growth, along with metabolism, disease resistance, and stress responses, by modulating gene expression [40].

### 3.3. α-Amylase Inhibition

The percentage level of α-amylase inhibition caused by the ethanolic extracts of the various different components of *B. ramiflora* is presented in Table 3. It was confirmed that the extent of enzyme inhibition rose as the concentrations of the *B. ramiflora* extracts increased. However, the highest levels of α-amylase inhibition for all concentrations were recorded for acarbose. When tested at 0.1–0.25 mg mL^−1^, the peel extract, however, produced significantly greater α-amylase inhibition than the flesh with seed, stems, stem bark, and leaves, respectively. The 1.0–2.5 mg mL^−1^ extracts from flesh with seeds of *B. ramiflora* provided α-amylase inhibition which was significantly greater than was the case for peel, stems, stem bark, and leaves, respectively. Moreover, the leaves and stem bark delivered the lowest levels of α-amylase inhibition, failing even to reach 50%.  Table 4 shows the IC_50_ measurements for all of the various *B. ramiflora* part extracts in the context of α-amylase inhibition. The IC_50_ measurements for all *B. ramiflora* samples exceeded those of the positive control, acarbose. For α-amylase inhibition, the IC_50_ values lay in a range from 0.50 ± 0.03 to 73.31 ± 12.16 mg mL^−1^ while for acarbose the IC_50_ measurement was 0.19 ± 0.04 mg mL^−1^. The α-amylase inhibition for the extracts of flesh with seed and peel produced IC_50_ values of 0.50 ± 0.03 and 0.61 ± 0.04 mg mL^−1^ which revealed no significant difference. For stems extracts, the IC_50_ measurement was 0.89 ± 0.11 mg mL^−1^, whereas for leaves there was a higher IC_50_ value at 73.31 ± 12.16 mg mL^−1^. Concurrently, it was revealed *B. racemosa* leaf extract comprises mild to moderate antidiabetic characteristics, which was previously investigated using in vitro antidiabetic assay, such as the α-amylase inhibition method. The methanol and ethanol leaf extracts of *B. racemosa* were observed to demonstrate comparable effectiveness in terms of antidiabetic activity, with IC_50_ values of 67.63 ± 0.36, and 67.46 ± 0.23 ppm, respectively [41]. The α-amylase helps to digest glycogen and starch and is secreted by the pancreas and salivary glands. Certain plants with medicinal properties have been observed to inhibit the activity of α-amylase. The plant-derived compounds, alkaloids, glycosides, galactomannan gum, polysaccharides, hypoglycans, peptidoglycans, guanidine, steroids, glycopeptides, and terpenoids, exhibit bioactivity to counteract hyperglycemia [42, 43].

### 3.4. α-Glucosidase Inhibition

For α-glucosidase inhibition, the results show that when the concentration is 0.1–2.5 mg mL^−1^, the different extracts from the various parts of *B. ramiflora* showed significant differences as can be observed from the findings in Table 5. For each of the extracts, the enzyme inhibition was shown to be dependent on the extract concentration used. For all concentrations tested, the greatest level of α-amylase inhibition was recorded for acarbose. When tested at 0.25–2.5 mg mL^−1^, the peel extract produced significantly greater α-glucosidase inhibition than was the case for flesh with seed, stems, stem bark, and leaves, respectively. The extracts of flesh with seed, stems, and peel produced nonsignificant differences in α-glucosidase inhibition at 0.1 mg mL^−1^, however. As the extract concentrations increased, α-glucosidase inhibition also rose as a percentage for each of the extracts.  Table 4 presents the IC_50_ measurements for the inhibition of α-glucosidase, revealing that each of the *B. ramiflora* extracts produced a higher IC_50_ value than was the case for acarbose. For the extracts, the IC_50_ measurements were from 0.59 ± 0.01 to 15.65 ± 0.95 mg mL^−1^, whereas for acarbose the IC_50_ value measured 0.10 ± 0.01 mg mL^−1^. The α-glucosidase inhibition of the extracts of peel, and flesh with seed produced IC_50_ values of 0.59 ± 0.01 and 0.69 ± 0.01 mg mL^−1^ which did not differ significantly, while for stems, the IC_50_ measurement was 0.99 ± 0.01 mg mL^−1^. Finally, IC_50_ values for the stem bark and leaves measured 3.69 ± 0.05 and 15.65 ± 0.95 mg mL^−1^, respectively. Plant-based extracts play a significant role as α-glucosidase inhibitors, emphasizing their potential in managing glucose levels. This inhibitory effect is primarily linked to the presence of phytoconstituents, including flavonoids, alkaloids, terpenoids, anthocyanins, glycosides, and phenolic compounds [44, 45]. The high phenolic content in the peel, and flesh with seed extracts likely accounted for their formidable inhibitory influence. A substantial influence on the obstruction of α-amylase and α-glucosidase was exhibited by the total phenolic content. Obtained from plant sources, natural inhibitors offer the advantage of inhibiting α-glucosidase. Furthermore, they may serve as an effective means of safely addressing postprandial hyperglycemia.

### 3.5. GC-MS Analysis

**Figure 1:**
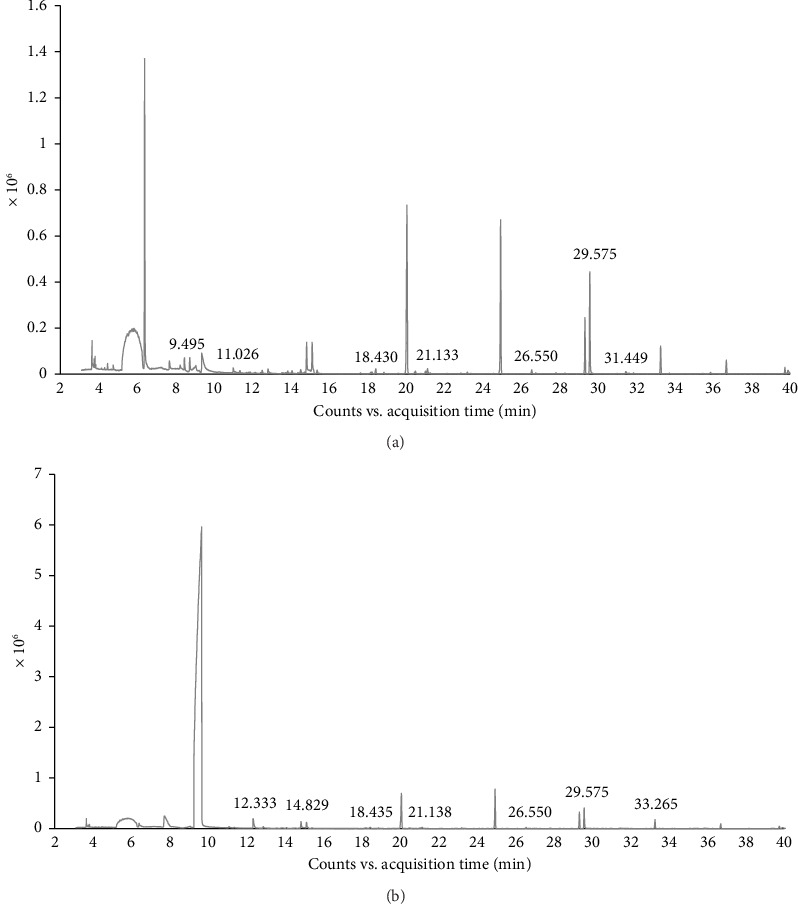
GC-MS chromatogram of *B.ramiflora*. (a) Flesh with seed extract; (b) peel extract.

The ethanolic extracts of *B. ramiflora* flesh with seed, and peel were analyzed using GC-MS to determine their phytochemical components. The GC-MS results revealed seven compounds in the flesh with seed sample (Table 6 and Figure 1(a)) and seven compounds in the peel sample (Table 7 and Figure 1(b)). Based on area percentage, major compounds present in the flesh with seed extract were identified as: phenol,3,5-bis(1,1-dimethylethyl)-, dimethylsulfoxonium formylmethylide, and methyl salicylate. The chromatographic analysis identified several compounds that may contribute to biological processes. Among these, phenol, 3,5-bis(1,1-dimethylethyl), a natural product derived from plant sources, has been studied for its potential as an anticancer agent [46] and antioxidant attributes [47]. Concerning the progression of insulin resistance and type 2 diabetes, oxidative stress has a substantial influence. Dimethylsulfoxonium formylmethylide is known for its antimicrobial properties [48]. Methyl salicylate, a volatile compound produced by plants, plays a significant role in signaling processes related to systemic acquired resistance and is involved in defense mechanisms against pests, microbial pathogens, and antagonists [49]. By suppressing inflammation and improving insulin function, methyl salicylate, a byproduct of salicylate, has demonstrated its ability to help those with type 2 diabetes achieve better glycemic control and decrease their blood sugar levels [50]. Table 7 indicates that the major compounds present in the peel extract are 2,4-di-tert-butylphenol, dimethyl sulfone, and cyclooctasiloxane, hexadecamethyl. Meanwhile, 2,4-di-tert-butylphenol was investigated for its antioxidant and anti-inflammatory activity [51]. Dimethyl sulfone, a naturally occurring organic sulfur compound found in various fruits, vegetables, grains, animals, and humans, is known to enhance the body's ability to produce insulin and promote carbohydrate metabolism [52] Moreover, cyclooctasiloxane, hexadecamethyl exhibits antibacterial activity [53]. As discovered by GC-MS, the recognition of bioactive combinations has signified that the effectiveness of *B. ramiflora* flesh with seed, and peel extracts in hindering α-amylase and α-glucosidase could be related to the existence of these bioactive combinations, which are acknowledged as having antidiabetic as well as several other pharmacological behaviors.

## 4. Conclusions

This research presents results which confirm that the ethanolic extracts of *B. ramiflora* may serve as sources of phenolic and flavonoid contents. The highest levels of α-amylase and α-glucosidase inhibition were demonstrated by the extracts of peel, and flesh with seed from *B. ramiflora*. These extracts proved more effective than the extracts from the stems, leaves, and stem bark of *B. ramiflora*. GC-MS revealed that various components in the ethanolic extracts of flesh with seed and peel may be involved in biological processes. It can therefore be concluded that *B. ramiflora* offers significant potential as a source of components which might find useful roles in nutraceuticals, functional foods, and as alternative treatments which might effectively serve to manage diabetes.

## Figures and Tables

**Table 1:** Total phenolic and flavonoid contents.

*B. ramiflora* extracts	Total phenolic content (mg GAE g^−1^ extract)	Flavonoid content (mg rutin g^−1^ extract)	Flavonoid/phenolic ratios
Leaves	163.89 ± 26.73^a^	62.55 ± 3.11^a^	0.38
Stems	12.33 ± 1.59^b^	6.58 ± 1.43^d^	0.53
Stem bark	15.10 ± 3.42^b^	6.99 ± 1.89^d^	0.46
Flesh with seed	145.83 ± 13.81^a^	23.46 ± 2.47^b^	0.16
Peel	155.73 ± 11.77^a^	13.17 ± 2.57^c^	0.085

*Note:* Each value is presented as mean ± SD (*n* ± 3). Mean within the column with different letters (a–d) shows statistically significant differences (*p* ≤ 0.05).

**Table 2:** Phytochemical constituents of *B. ramiflora*.

*B. ramiflora* extracts	Phytochemical constituents						
	Steroids	Flavonoids	Tannins	Terpenoids	Anthraquinones	Reducing sugars	Saponins
Leaves	−	+	+	+	−	+	−
Stems	−	+	−	+	−	+	−
Stem bark	−	+	−	+	−	+	−
Flesh with seed	−	+	+	+	−	−	−
Peel	−	+	+	+	+	−	−

*Note:* (−) negative test; (+) positive test.

**Table 3:** α-Amylase inhibition effect of *B. ramiflora* extracts at different concentrations (0.10–2.5 mg mL^−1^).

*B. ramiflora* extracts	α-Amylase inhibition (%)				
Concentration (mg mL^−1^)	0.10	0.25	0.50	1.0	2.5
Acarbose	45.10 ± 2.58^aE^	52.72 ± 1.10^aD^	56.46 ± 1.07^aC^	61.34 ± 0.54^aB^	70.14 ± 1.06^aA^
Leaves	1.83 ± 0.91^eE^	7.11 ± 0.74^fD^	15.01 ± 0.97^eC^	18.08 ± 1.23^eB^	25.13 ± 0.86^eA^
Stems	35.09 ± 0.99^cE^	38.95 ± 0.56^dD^	46.20 ± 0.88^cC^	51.98 ± 1.08^cB^	57.02 ± 0.97^cA^
Stem bark	8.23 ± 0.11^dE^	17.05 ± 0.79^eD^	25.47 ± 0.32^dC^	30.18 ± 0.73^dB^	41.44 ± 0.05^dA^
Flesh with seed	36.37 ± 0.25^cD^	42.25 ± 0.51^cD^	49.38 ± 0.78^bC^	57.18 ± 1.06^bB^	64.61 ± 0.82^bA^
Peel	39.31 ± 0.14^bD^	45.09 ± 0.82^bD^	48.12 ± 0.23^bC^	53.24 ± 0.45^cB^	58.24 ± 0.37^cA^

*Note:* Each value is presented as mean ± SD (*n* = 3). Mean within the column with different letters (a–f) shows significant statistical differences (*p* ≤ 0.05). Mean within the row with different letters (A–E) shows significant statistical differences (*p* ≤ 0.05).

**Table 4:** IC_50_ (mg mL^−1^) value of *B. ramiflora* extracts on α-amylase and α-glucosidase inhibition.

*B. ramiflora* extracts	IC_50_ (mg mL^−1^)	
	α-Amylase inhibition	α-Glucosidase inhibition
Acarbose	0.19 ± 0.04^e^	0.10 ± 0.01^e^
Leaves	73.31 ± 12.16^a^	15.65 ± 0.95^a^
Stems	0.89 ± 0.11^c^	0.99 ± 0.01^c^
Stem bark	6.13 ± 0.21^b^	3.69 ± 0.05^b^
Flesh with seed	0.50 ± 0.03^d^	0.69 ± 0.01^d^
Peel	0.61 ± 0.04^d^	0.59 ± 0.01^d^

*Note:* Each value is presented as mean ± SD (*n* = 3). Mean with different letters (a–e) shows significant statistical differences (*p* ≤ 0.05).

**Table 5:** α-Glucosidase inhibition effect of *B. ramiflora* extracts at different concentrations (0.10–2.5 mg mL^−1^).

*B. ramiflora* extracts	α-Glucosidase inhibition (%)				
Concentration (mg mL^−1^)	0.10	0.25	0.50	1.0	2.5
Acarbose	50.19 ± 1.54^aE^	62.97 ± 2.07^aD^	74.92 ± 1.85^aC^	82.03 ± 2.02^aB^	95.09 ± 1.97^aA^
Leaves	7.11 ± 0.02^dE^	16.12 ± 0.36^fD^	21.93 ± 0.10^fC^	27.15 ± 0.30^fB^	34.19 ± 0.47^fA^
Stems	13.18 ± 0.25^bE^	25.08 ± 0.21^dD^	37.25 ± 0.09^dC^	51.19 ± 0.14^dB^	66.05 ± 0.29^dA^
Stem bark	9.18 ± 0.10^cE^	18.21 ± 0.36^eD^	25.15 ± 0.21^eC^	35.30 ± 0.87^eB^	46.14 ± 0.31^eA^
Flesh with seed	13.56 ± 0.43^bE^	29.14 ± 0.17^cD^	42.03 ± 0.51^cC^	56.05 ± 0.22^cB^	77.91 ± 0.63^cA^
Peel	12.61 ± 0.58^bE^	32.22 ± 0.39^bD^	45.35 ± 0.08^bC^	59.41 ± 0.34^bB^	82.60 ± 0.34^bA^

*Note:* Each value is presented as mean ± SD (*n* = 3). Mean within the column with different letters (a–f) shows significant statistical differences (*p* ≤ 0.05). Mean within the row with different letters (A–E) shows significant statistical differences (*p* ≤ 0.05).

**Table 6:** Chemical profile of *B. ramiflora* flesh with seed extract by GC-MS.

Peak	Retention time (min)	Compounds	Molecular weight	Chemical formula
1	9.495	Dimethylsulfoxonium formylmethylide	120.17	C_4_H_8_O_2_S
2	11.026	Phenol, 2,6-dimethyl-4-[[5-(4-pyridinyl)-1H-1,2,3,4-tetrazol-1-yl]methyl]-	281.31	C_15_H_15_N_5_O
3	18.430	Propylphosphonic acid, fluoroanhydride, 4-methylcyclohexyl ester	222.23	C_10_H_20_FO_2_P
4	21.133	Methyl salicylate	152.14	C_8_H_8_O_3_
5	26.550	Trans-2-methyl-4-n-pentylthiane, S,S-dioxide	218.35	C_11_H_22_O_2_S
6	29.575	Phenol, 3,5-bis(1,1-dimethylethyl)-	206.32	C_14_H_22_O
7	31.449	Trans-2,4-Dimethylthiane, S,S-dioxide	162.24	C_7_H_14_O_2_S

**Table 7:** Chemical profile of *B. ramiflora* peel extract by GC-MS.

Peak	Retention time (min)	Compounds	Molecular weight	Chemical formula
1	12.333	Dimethyl sulfone	94.13	C_2_H_6_O_2_S
2	14.829	Tetraethyl silicate	208.32	C_8_H_20_O_4_Si
3	18.435	Propylphosphonic acid, fluoroanhydride, 4-methylcyclohexyl ester	222.23	C_10_H_20_FO_2_P
4	21.139	Methyl salicylate	152.14	C_8_H_8_O_3_
5	26.550	Trans-2,4-Dimethylthiane, S,S-dioxide	162.24	C_7_H_14_O_2_S
6	29.575	2,4-Di-tert-butylphenol	206.32	C_14_H_22_O
7	33.265	Cyclooctasiloxane, hexadecamethyl-	593.23	C_16_H_48_O_8_Si_8_

## Data Availability

The data that support the findings of this study are available from the corresponding author upon reasonable request.
